# Exploratory study of qualitative psychological factors influencing performance in esports

**DOI:** 10.3389/fspor.2025.1505409

**Published:** 2025-05-22

**Authors:** Dávid Papp, Réka Burján, Csilla Csukonyi

**Affiliations:** Faculty of Humanities, University of Debrecen Institute of Psychology, Debrecen, Hungary

**Keywords:** esports, performance, interview, focus group, Atlas.ti

## Abstract

**Introduction:**

A comprehensive understanding of the psychological characteristics underlying performance in esports is key to the effectiveness of sport psychology work. In the present research, we aimed to identify the psychological factors that influence the performance of e-athletes and to confirm the results of previous research on specific characteristics.

**Methods:**

The study used a qualitative method consisting of focus group discussions (*N* = 15) and semi-structured interviews (*N* = 30). We applied content analysis using Atlas.ti content analysis software with the assistance of three independent coders.

**Results:**

The results suggest that the most significant characteristic behind the performance of e-athletes is their resilience and resilient personality. In addition, generalisable traits such as stress tolerance and sustained attention also had high-frequency values. However, some characteristics are specific to the eSport genre as well as to the particular eSport title of individual or team esports with varying emphasis on such things as cooperation and communication skills. The characteristics used in the analysis were classified into six groups of competencies.

**Discussion:**

Finally, as a result of the research, it was possible to identify characteristics and classify them into competencies, which can serve as a reference point in the field of selection and talent management and can also serve as a basis for the development of psychodiagnostic measurements and tests for e-athletes in the future.

## 1 Introduction

Esports are gaining ground both nationally and internationally, as there is an estimated 100 million worldwide growth in spectators from 2022 to 2025 (1), and the League of Legends World Championship peak viewership was 6,86 million in 2024 (2), furthermore, the esports market revenue is estimated to reach 5.93 billion U.S. dollars by 2029 (3). However, this growth is not only for spectators and players, as the academic world has also started to show increased interest in the topic of playing video games at a competitive level. One of the most commonly cited definitions of esports is that of Wagner, who defines esports as “an area of sports activity in which people develop and exercise mental or physical skills using information and communication technology” [(4), p. 3]. Esports could also be labelled as mental sports (5), and the definition given earlier confirms that mental abilities and cognitive skills (6, 7) are involved in the activity. On this basis, there has to be a set of measurable psychological characteristics that can potentially influence performance during esports.

### 1.1 Esports genres and sub-genres

**Figure 1:**
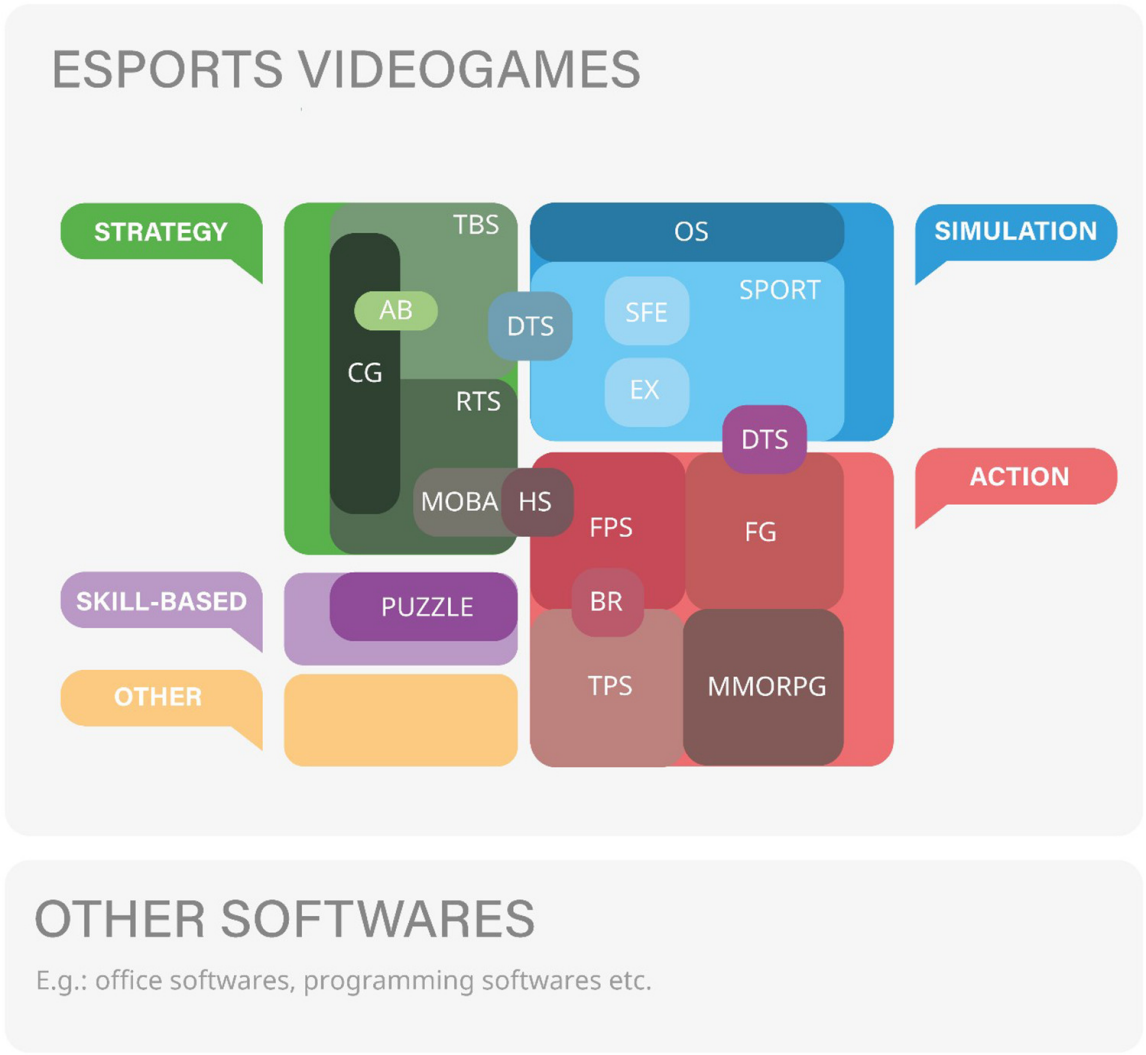
Esports genres and subgenres [based on (9–22), own editing].^1^

Esports is a collective term that encompasses a wide range of game genres and titles. The most prominent esports game genres include first-person shooters (FPS), multiplayer online battle arenas (MOBA), real-time strategy games (RTS), sports simulation games, card games and fighting games (8). However, there are many more genres represented in the esports scene, so we have attempted to draw on existing video game classification systems (9–11) and oher video game and esports-specific literature (12–22) to bring together in a comprehensive diagram the video game genres and subgenres from which official competitions are organised (Figure 1).[Fn FN0001]

In terms of genres, the first major cluster is action video games, with a strong emphasis on fast movement and continuous action and events. Shooting, fighting and action role-playing games can be grouped into this category (9–11, 15, 20). Perhaps one of the best-known genres in the shooting game category is the First Person Shooter games. Within these, most of these games that are considered esports are team-based tactical games, where team sizes typically range from four (e.g., Call of Duty), five (e.g., Counter-Strike, Paladins, Tom Clancy's Rainbow Six Siege and Valorant) and six (e.g., Overwatch), depending on the game mode. In these video games, the player cannot see their characters in their entirety and can only see their arms and weapons in the basic view, hence the first person term for the genre. The games usually involve two teams playing against each other using the tools provided by the game (10, 11, 15, 18, 20).

It is also worth mentioning that there are also shooting games that use a third-person external view (Third Person Shooter, TPS) instead of an internal, first-person view. An esports title example of this genre is Splatoon. Combining elements of FPS and TPS games, the Battle Royale genre (e.g., Apex Legends, Fortnite and PlayerUnknown's Battlegrounds) has been created, which can be played solo or in teams. An important difference from the previous ones is that the player has to deal with a large, freely explorable world, while in traditional FPS and TPS games, there is usually a much smaller area available. The large area allows up to 100 players to be in a match at the same time, but the large size also means that after a time limit, the playable area of the world begins to shrink, and players are compelled to find each other. Another difference is that weapons and other equipment are not given or bought by the player, but they are scattered around the map, and players have to find these to increase their chances of winning (10, 11, 18, 20).

Action video games also include fighting games, which are mainly single-player esports games. The player controls a character of their choice (in some games, players can team up by choosing multiple characters, but even if they can switch between them during the fight, they only fully control one character at a given time) in an arena, which is generally smaller in area thus most of the information of what is happening can be monitored on the screen. Time limits are also a significant factor in most games, affecting the outcome of the match. Fighting games could be further differentiated, according to whether they are digital versions of real fighting sports (e.g., EA UFC and WWE 2K) or fantasy games (e.g., Dragon Ball FighterZ) and whether they are two-dimensional (e.g., Mortal Kombat, Super Smash Bros and Street Fighter) or three-dimensional (e.g., For Honor, Tekken) (10, 11, 18, 20).

The last eSport game genre subgroup of action video games is Action Role-playing games, where character customisation and the story from that character's point of view are an important factor. However, mostly massively multiplayer online role-playing games (MMORPGs) can be classified as esports. World of Warcraft is an adequate example because it is one of the best-known games in the genre and it offers two modes for e-athletes. The first and longest-established mode is called Arena, in which two- or three-player teams compete in an in-game arena. The second mode is a much more recent initiative called Mythic Dungeon International. In this mode, teams of five compete to overcome challenges in the shortest possible time and with the fewest number of mistakes, and then the results are used for comparison. In both modes, teams will be tested on their knowledge of their character and the game, as well as their teamwork, just in different ways either Player vs. Player or Player vs. Environment (9–11, 18, 20).

Separating the group of strategic games can be a dividing point, as it is undeniable that all esports games require some level of strategy and strategic thinking. However, in strategy games, it is as if a military operation is not only planned but also executed, so strategy and tactics are crucial from the very beginning. In most games, you can see what is happening from a bird's eye view, and the camera often allows free control independent of the base or character, so that players can gather as much information as possible. It includes the genre of Real-Time Strategy Games (RTS) (e.g., Age of Empires, Starcraft and Warcraft), where the events in the game happen in real-time, i.e., continuously and simultaneously, thus making time and resource management a fundamental aspect. In general, the gameplay is that the player chooses a nation or race (in the case of fantasy and science fiction games) at the start of the game, taking into account all its strengths and weaknesses, then, as a warlord or ruler, builds a base and manages resources to assemble an army as quickly as possible to defeat the base built by the other player or players (9–11, 18, 20).

Multiplayer Online Battle Arenas can be considered a subcategory of real-time strategy games, mainly because they were initially based on these games (notably Warcraft 3). Dota 2 and League of Legends are the most prominent examples. Such games are unique within RTS games, as instead of an army, the player controls a specific character (and a specific role) within a typical team of five. Two teams compete to see who can destroy their opponent's base the fastest, aided by the game's particular abilities, items, watchtowers and AI-controlled creatures (11, 14, 18).

The second subgenre of strategy games is Turn-based strategy (TBS), which differs from RTS in that the events in the game are sequenced in turns, giving players time, albeit sometimes limited, to think through the options and plan their moves. Civilization could be a typical example (9–11, 20).

Autobattler games are a subgenre within turn-based strategy games. This is the latest genre of strategy games, but there are eSport game examples like Hearthstone Battlegrounds and Teamfight Tactics. From the gameplay perspective, the players have a say in the game only during the limited-time preparation phase, when they have to make tactical decisions, after that, the second half of the round, the fight itself, is played automatically, which the player cannot influence and then the next round starts. In the preparation phase, the game randomly offers options (in the form of characters, cards, objects, etc.), where the choice can be influenced by the type or category they fall into, as they have synergies and typically perform better together (13).

A larger group of eSport video games are (digital collectable) card games, which for this categorisation we considered as part of the strategy games category, as they are based on tactical and strategic thinking (19). Hearthstone and Magic The Gathering: Arena are typical examples. The focus is on those tactics and strategies players use to build their decks before each match from the cards they have available (which can be characterised by different attributes, strengths and abilities). The players then compete against their opponent's deck in a typically turn-based game, where the outcome is influenced by the card draw odds. The various eSport card games are designed by the game developers so that there are no “perfect” decks, but decks with both strengths and weaknesses, where the weaknesses can be minimised with the right knowledge and strategy (11, 19).

Simulation games are specifically designed to simulate a given situation or activity as accurately as possible. Within this category, there is one completely separate genre, what we call Other Simulation Games. It is an umbrella term, as games that simulate non-sporting activities, but still have a competitive scene are diverse. Demonstrating this through two examples: Farming Simulator 23, which in terms of gameplay briefly ranges from choosing adequate agricultural vehicles to harvesting and delivering them as a team. Another example is the life simulator The Sims 4, which has also been the subject of competitions, where teams of three (architect and interior designer, designer of the people living in the apartment and storyteller) have tested their creativity (9–11, 23, 24).

Sports games form a larger subgenre, as they aim to simulate as accurately as possible the sporting activity that the game is based on (9–11). This category is also diverse, as it includes not only video games based on real-life sports as (1) Digital traditional sports (e.g., EA Sports FC, NBA 2K, Gran Turismo and Formula 1) but also (2) Sports games with fantasy elements (e.g., Rocket League, Mario Kart and Mario Tennis) and (3) Exergames (e.g., Just Dance and Zwift). The first subcategory aims to accurately simulate the traditional sports on which they are based, mainly in terms of rules and options, with players mostly choosing a team and controlling a virtual athlete at a time from an external viewpoint or first-person view in the case of motorsports. The second subcategory is also inspired by traditional sports, but with the addition of gaming elements primarily intended for entertainment and spectacular gameplay, which could not be reproduced in reality, but in terms of their gameplay they are relatively similar to the first subcategory. Finally, the fundamental purpose of the third subcategory was the gamification of physical movement, allowing players to actively use sensors and sports equipment (such as a smart indoor bike in the case of Zwift) to exert an effect in the game and thus measure themselves in virtual space by moving their entire body (11, 17, 20, 22).

The genre of Skill and Logic games is highlighted because it includes the Puzzle games subgenre. These are typically tile-matching games, where the aim is to match the tiles offered in the correct order, in the right place, where speed usually gives an advantage and more points, which can later be compared with the scores of other players. It includes various adaptations of the well-known Tetris game (e.g., Tetris 99), as well as Puyo Puyo, which is similar in function to Tetris but with a greater emphasis on the similarity of tiles during gameplay (10, 11, 16, 21).

The last genre group we called Other. These esports games can not be classified in the previous genre categories based on gameplay. For example, Geoguessr, a browser-based geo-game where players have to guess the exact location of a place using Google Street View images, i.e., where in the world they would see this. The more accurate and faster the guess, the more points the player scores (11, 12).

When presenting the esports genres, it is also important to note that, thanks to innovative ideas and a combination of different game elements, some genres do not fit into a single set, but can be seen as a cross-section of several genres (11). It includes the so-called hero shooter genre, which combines elements of MOBA and FPS games (e.g., Overwatch and Valorant). The gameplay is similar to an FPS game but includes fantasy elements, as instead of just weapons and tools, players in addition choose characters that have abilities and play different roles accordingly (11, 18). Some card games, such as Clash Royale, have additional strategic elements, such as base protection, through the skills of the cards and combat units (25). Moreover, taking into account the typical game elements of turn-based strategy games, most card games, as well as sports games such as chess, could fit the criteria of the genre (26).

In addition to the video game genres included in esports, it is worth looking at the segments not based on video games but implemented using other software. These competitions are also typically included under the umbrella term electronic sports, examples include Microsoft Excel and programming competitions (27, 28).

Therefore, esports can be rather diverse and a wide range of skills are required for an e-athlete to reach peak performance in the given subgenre.

### 1.2 Qualitative research on skills related to esports performance

Previous research using a qualitative methodology has specifically focused on esports, and e-athletes have aimed to explore different themes and issues compared to the focus of the present paper. These include the idea that online games (e.g., Starcraft, Minecraft or World of Warcraft) through the power of community and gaming environment could provide reinforced circumstances and have a positive impact on the language learning process (29–35). Game elements specific to esports could also be transferred through gamification into education, where they could have a positive impact by sustaining motivation over time (30, 36–40). Exploratory research has also covered the social inequalities in the esports sector that can hinder efforts towards inclusion (41, 42). An important aspect highlighted in previous qualitative research is the process of becoming an e-athlete and the characteristics behind the career choice that may motivate or even discourage players from pursuing a competitive career (43–45). In addition, the potential positive and negative effects surrounding this career have also been identified (46).

The research that aimed to identify and explore the characteristics that can influence performance examined one or two mental, cognitive and/or social skills and abilities specifically. It can be exemplified by the impact on the performance of knowing and using digital communication features, such as the challenges of online communication and the appropriate use of in-game chat and ping functions for callouts, and also use communicational tools for interpersonal conflict resolution (41, 47–52, 75). A closely related theme is the importance of acquiring game-specific interactions and skills, basic game knowledge and other tactical and strategic elements that can underlie differences in performance levels in the game (37, 53–57). Anticipation is based on knowledge of the game; thus, situational awareness and insight have also been explored earlier as a function of performance (53, 58). With a focus on team esports, the characteristics and challenges of digital collaboration have also provided a typical research topic, including team characteristics that affect performance in joint action, such as team cohesion, congruence, and diversity, i.e., the cohesion that is essential to achieve synergy (48, 50, 54, 75). Cooperation and adaptability have also been investigated as essential factors for teamwork (49, 56, 59–61). The in-game leader role is often experienced in team games, where the leadership competencies associated with this role are closely related to team mood and team performance (62, 75). Motor skills, such as hand-eye coordination and related reaction times, are highly emphasised in the context of esports performance, even in qualitative methodological research (58, 63), and within cognitive abilities, attentional features such as sustained attention, attentional control and eye movement (48, 76), and performance degradation due to attention sharing (64) also received prominent scientific attention. Finally, the challenges and failures experienced during competition and the esports career as stressors,coping with stress in the short term and under pressure stress management (48, 52, 65), also the long-term management and positive framing of these and thus resilience (48, 66), moreover control over thoughts, goals and behaviour (48) were linked to performance in esports. However, several of these studies could be characterised by only applying a moderately small sample size and investigating just one esports title [such as (53, 65, 66)].

In summary, previous research suggests that performance in esports is influenced by characteristics such as adaptability, the ability to cooperate with others, sustained attention and the ability to share attention, good memory linked to knowledge of the game, the ability to communicate within the digital space, eye-hand coordination, fast reaction time, stress tolerance and resilience.

The aforementioned research has provided valuable insights into specific segments of esports, but was limited by sample size and focused on one specific esports title. Building on this, the present research aims to confirm the psychological factors previously investigated as well as to identify new potential underlying factors behind the performance of esports in general and, through that, achieve a deeper understanding with qualitative insight. On this basis, our research questions were the following:

1. What are the mental factors that could influence performance in esports?
2. To what extent can the factors, which were identified in previous qualitative research, be generalised (regardless of the esports title)?
3. Are there any general mental characteristics that have been less or not at all addressed in previous qualitative research?
4. How do the factors identified relate to each other?
  4.1 Is it possible to group them?

## 2 Materials and methods

### 2.1 Measures

The semi-structured interview frameworks used for the focus group discussions and the interviews were based on the work of Himmelstein et al. (66) and Csukonyi et al. (67). The focus group discussions took about 1 h per group, while the interviews took about 1.5 h per participant. Participants had to read and electronically sign a consent form before participating in the study.

The focus group discussion consisted of three main sections and topics: (1) A conversation about video games and esports in general, where we asked questions such as “Please tell us in a few sentences how your life as a “gamer” started, what and why you got involved in this “world"?”. (2) Exploring the psychological factors of esports, which included questions best suited to the research topic, such as “If you had to name the mental characteristics that most or only the best players have, what would they be?”. (3) The links between sport and performance psychology and the development of experiences, during which we asked leading questions such as “Think back to a time or situation when you played particularly well! What was it like? How did it feel to experience that? What do you think led to that experience?”.

During the interview, we first asked demographic questions and asked about how they were connected to the world of esports. After that, the first main topic was the background of the involvement in esports (e.g., “When did you start playing esports?”). Next, we asked about esports and video games (e.g., “What is your main e-sports game?”), and then we looked at achievements and goals (e.g., “What do you think has made you successful?”). The exploration of psychological characteristics was the most comprehensive theme, with questions covering six subthemes, such as the mental strengths and characteristics behind success (e.g., “What do you think is your greatest strength, positive trait that makes you stand out from your eSporting peers? “); the subjective experience of good performance (e.g., “Think back to a situation or time when you played particularly well, what was it like, how did it feel and what led to that experience?”); challenges, obstacles and motivating factors (e.g., “What are the mental challenges and obstacles you face when competing?”); mental preparation (e.g., “Tell me about the methods and techniques you use to prepare for a competitive situation!”); teamwork characteristics (e.g., “If you work in a team, do you work with a specific team or do you join a randomly selected team during your game?”); and goal completion (e.g., “Do you prefer to achieve your goals alone or with the help of partners at team level?”). As a prelude, we asked two questions, one related to the relationship between sports psychology and esports (“In what various ways do you think a sports psychologist could help gamers and e-athletes like you?”), and the other on dual career planning for e-athletes and post-athletics plans (“In what field could you see yourself most after finishing your e-sports career?”). These questions were deemed sufficient to confirm and also identify psychological factors that could affect performance, also, these were formulated in a more general manner to be of use in any esports title.

To ensure qualitative rigor, we considered both data saturation and researcher reflexivity throughout the study. The snowball method organically circled back to individuals from previously contacted networks, suggesting that we had reached the core of our intended population. This, along with the repetition of key themes in the later stages of data collection, indicated that thematic saturation had been achieved. In addition, the research team maintained reflexivity through regular discussions to minimize bias and ensure balanced interpretation.

### 2.2 Participants

For this research, we applied the snowball sampling method. The data were collected by online video calls from September 2021 to July 2022. The focus group interviews were conducted with 15 participants divided into 4 groups (3-3-4-5), while the interviews were conducted with 30 participants. In terms of the characteristics of the subsample that participated in the focus group interviews, 13 of the participants were male, and the average age was 23.7, with a range of 5, a minimum of 18 and a maximum of 37. Our focus group interview subjects included 10 professional e-athletes, 2-2 coaches and researchers, and one team owner/manager.

Among the subsample of interviewees, 27 of them were male, the average age was 25, with an age range of 4.3, a minimum of 18 and a maximum of 34. This subsample included responses from 20 professional e-athletes, 4 researchers, 3 coaches, 2 event organisers and 1 commentator. The total sample thus consisted of 45 people (40 of them men, with a mean age of 24.6, an age standard deviation of 4.5, a minimum of 18 and a maximum of 37). The participants represented 15 esports titles^2^.

The professional e-athletes were all certified players of Hungarian esports organizations, and achieved results in international and top-tier national competitions and/or achieved high places in the ranked system of their chosen esports title. The average years of experience was 5,42 (SD = 2,63). Out of these 30 professional e-athletes, 22 reported having most of their experience and results in team esports. Among these 4 reported to be their team's in-game leader or IGL in short (3 tank and one support player), the others preferred roles were 9 damage dealers, 2 tanks, 3 supports in MOBAs and Hero-shooters, and 3 fraggers and one sniper in Shooter games.

### 2.3 Data analysis method

The total content of the focus group and interviews was 523 pages, comprising 1652284 characters. Three independent coders were involved in the content analysis, based on predefined codes. In the content analysis, the codes corresponded to psychological characteristics. Atlas.ti content analysis software was used for the content analysis (68).

The content of the focus group discussions was used beforehand to identify and subsequently code psychological characteristics that have not been previously explored in any or little research on the relevance of esports in the overall content analysis. This process involved 28 psychology graduate students. Drawing on their subject knowledge, they were asked to independently extract psychological concepts and characteristics from the transcripts, mark relevant references, and count their frequency of occurrence. All these were summarized later, taking into account the conceptual synonyms and the rules of suffix morphology^3^ and verb conjugation resulting from the specificity of the Hungarian language, keeping in mind the frequencies. To ensure consistency, these individual data sets were compared and harmonized through a synthesis process, during which overlapping or synonymous items were merged, and divergences were resolved via consensus among the research team. The most frequently mentioned and commonly agreed-upon concepts were retained. These student-derived inputs served two main functions: (1) they confirmed the relevance of codes already identified in the existing literature, and (2) they expanded the coding scheme by contributing new psychological characteristics that were both frequent and collectively endorsed. All of these codes used for the content analysis, along with the keywords used for the analysis in ATLAS.ti, are presented in Table 1.

**Table 1:** Search words for codes.

Code	Applied search words^a^
Adaptability	Adapt, adaptability, adaptation, align, change, conform, flexible, new, patch, uncommon, unusual
Goal-orientedness	Achieve, ambition, ambitious, aspire, conscious, develop, do, goal, goal setting, goal-orientedness, implement, like to, strive, to make a difference, to make something come true, want, will
Decision making	Alternatives, choose, correct, correctly, decision, make, making a decision, opportunity, precise, precisely, relevant
Cooperation	Cohesion, collective, collectively, company, cooperation, cooperative, duo, each other, group, pair, premade, team, to fit in, together
Sustained attention	Ability to concentrate, attention, attention focus, concentrate, concentration, continuous, fast reaction, focus, fully, head, pay attention, react, sustain, to focus
Situational awareness	
and insight	Adapt, adaptability, anticipation, call, conclude, conclusion, conscious, counter, enemy, estimate, experience, feel, future, intent, intuition, know, logic, punish, recognise, situational awareness, situationally aware, timely, wait, what, where
Communication skills	Communicate, communication, contact, miss, ping, say, sign, signal, speak, ss, talk, together
Creative problem-solving	Count, creative, creativity, idea, original, problem, problem-solving, solving (problems), specific
Critical thinking	Analytical, analyze, assess, better, critic, critical, evaluate, logic, logical, reinterpret, thinking
Memory	Memory, memory capacity, memorize, mind, remember, something pops up (in mind)
Monotony tolerance	Always, bore, boredom, continuous, identical, monotone, monotony, monotony tolerance, non-stop, prolonged, protracted, same, tolerate
Reaction time	Button, button press, fast, fragment, immediately, keyboard, moment, muscle memory, pass, react, reaction, reaction time, reflex, right away, seconds, time
Resilience	A lot of time, ambition, ambitious, continuous, dedicated, determination, determined, endure, exercise, moving on, never giving up, not affected by, patience, perseverance, persevere, practice, recover (from a situation), survive, tired, want, will
Stress tolerance	air, balance, beat, block out, burnout, calm, composed, confusion, confused, difficult, excited, expectation, freeze, frustration, frustrated, heart, nerve, nervous, pressure, pulsate, self-control, stage fright, stress, sweat, tension, tense, thrill, work
Eye-hand coordination	Coordination, dexterity, eye, finger, handy, hand, hit, mechanical, miss, precise, shake, skill, twitching
Simultaneous attention capacity	Another, at the same time, many, much, more, parallel, share, shared, simultaneously, simultaneous, side by side, multitasking
Achievement motivation	Achievement motivation, compete, competition, competitive, competitive spirit, motivated, motivation, performance

^a^ According to the rules for the Hungarian language, the dictionary forms of verbs and nouns and their substituted or conjugated forms were also searched.

To ensure consistency and reliability in our content analysis, disagreements between coders were resolved by including only those segments that received mutual agreement. Disputed segments were excluded from the final analysis to maintain a conservative and consensual coding approach. The highest overall frequency of occurrence was for resilience (Sum.Gr = 863), while the lowest frequency was for simultaneous attention capacity (Sum.Gr = 86). Among the codes shared by all coders, the highest frequency was also for resilience, while the lowest frequency was for memory (Gr = 30). The total number of codes common to the three coders (Gr) was 3651. Agreement among coders was measured using the ATLAS.ti percent agreement index, the Holsti index and the Krippendorff c-Alpha binary. To calculate the overall agreement among coders, Krippendorff's c-Alpha binary value (0.569) and Krippendorff's Cu-Alpha value (0.433) were considered, as participants could refer to more than one code in a sentence. There was more than 50% agreement among coders on simultaneous attention capacity (54.9%), sustained attention (54.7%), reaction time (51.5%) and eye-hand coordination (51.2%). The percentage agreement was not less than a quarter of the total for any of our codes, with the lowest percentages of agreement falling below 37% for creative problem-solving and achievement motivation. The intercoder reliability values are illustrated in Table 2.

**Table 2:** Intercoder reliability values among coders.

Code name	Simple percent agreement	Holsti index^a^	Krippendorff's c-Alpha binary^b^	Gr.	Sum. Gr.
Simultaneous attention capacity	54.90%	61.90%	0.611	61	86
Sustained attention	54.70%	63.40%	0.635	362	472
Reaction time	51.50%	58.90%	0.586	156	204
Eye-hand coordination	51.20%	56.50%	0.558	94	141
Adaptability	46.80%	50.00%	0.493	142	237
Communication skills	44.80%	47.20%	0.485	179	293
Cooperation	44.10%	45.80%	0.456	426	692
Stress tolerance	43.40%	45.50%	0.458	479	723
Situational awareness and insight	42.40%	41.60%	0.407	252	441
Goal-orientedness	41.90%	41.60%	0.401	260	471
Memory	40.60%	36.20%	0.34	30	90
Resilience	40.40%	37.80%	0.373	532	893
Decision making	40.10%	35.80%	0.389	116	192
Critical thinking	38.90%	33.20%	0.33	176	364
Monotony tolerance	38.20%	30.60%	0.316	120	231
Creative problem-solving	37.70%	24.30%	0.239	81	179
Achievement motivation	37.00%	28.30%	0.289	185	425

Source: PTAE database.

^a^ Percent agreement between coders taking into account variation in data segments (71), cited in (72).

^b^ Of the codes/domains (comparing coded and uncoded domains), which codes are satisfactory in terms of the agreement among coders, and which ones are understood differently by different coders [(73), cited in (72)].

The research was conducted with the approval of the United Ethical Review Committee for Research in Psychology (EPKEB), taking into account relevant research ethical considerations. The ethical approval issued by the Committee has the reference number 2021-36.

## 3 Results

### 3.1 Identified psychological factors

As detailed above, the code with the highest frequency was that of resilience (Gr = 532), suggesting that this characteristic is the most supportive of e-athletes' sport performance and across their career. An extraordinary number of participants referred to the importance of perseverance in a critical competitive situation, or even in a prolonged challenging situation and the composure they often mentioned to keep going in the long run and to keep competing despite setbacks. In cases where respondents talked about resilience, they also usually mentioned stress tolerance (Gr = 479), which was the second most frequently mentioned. The participants mainly referred to this code as stress in competitive situations but also as frustration at making mistakes and in the face of negative criticism. In addition to talking about the level of stress they experienced, they often reported that stress management and stress tolerance was the key to success. As a significant part of e-sports is a team game (69), comments referring to cooperation were also frequent (Gr = 426). Team e-athletes have underlined the link between strategic and tactical teamwork and competitive performance. Many reported that the good atmosphere and strong collaboration among team members also boost performance.

We found that the cognitive skills most often mentioned by respondents during the interviews were sustained attention (Gr = 362). Participants repeatedly pointed out that sustained attention is the key to success in esports, where any disturbance or fundamental lack of concentration prevents players from reaching peak performance. Sixth place was given to goal-orientedness (Gr = 206), while seventh place was given to situational awareness and insight (Gr = 252). During the interviews, participants identified setting and following long-term goals as essential for a successful career in esports, which were mostly influenced by their intrinsic motivation. Situational awareness and insight were related to their in-game performance by thinking with the other player's head and predicting the future based on game mechanics. Nearly the same number of content was coded for achievement motivation (Gr = 185), communication skills (Gr = 179) and critical thinking (Gr = 176). Many highlighted that the heightened emotional state they experience during competitions has a positive impact on their performance, there were many references to actively striving to improve their skills and performance, and there was also much general talk about achievement motivation as a driving force that can fuel their long-term perseverance and resilience.

The ability to communicate has been identified as a significant factor in general as in communicating with the coach, but in the case of e-athletes playing in a team, it has been referred to as an almost indispensable characteristic. Nearly equal amounts of respondents reported the dominant influence of critical thinking. High-level e-athlete performance was considered unfeasible if the person did not review their performance and was unable to take constructive criticism. A significant proportion of e-sports titles, such as action games and MOBAs (69), require an immediate response reaction from players, so the next in numerical order was reaction time (Gr = 156). This was also frequently mentioned along with the eye-hand coordination ability (Gr = 94). However, in contrast to expectations and previous research findings, we found a lower prevalence of these codes, which was because participants took it for granted that high-performing e-athletes have the motor skills required for esports, and so did not make much of it, but they did draw differences along the lines that some esports titles may require different levels of these characteristics. They had a similar opinion about the simultaneous attention capacity (Gr = 61) [“*Yeah, well, of course, the basics, an e-athlete won't do much if they don't have the motor skills or if they can't actively pay attention to several things at the same time*.” (Female researcher, 24)].

A comment referring to adaptability was identified 156 times in our interviews. This term was used in two different ways by the participants. In one case, adaptation was understood to refer to things related to the game (e.g., new rule systems, game update changes) and to situational changes associated with esports activities (e.g., new teammates). In both interpretations, a high level of adaptability was seen as crucial for smooth development, but above all for staying in the sector. As in the life of every competitive athlete, e-athletes are challenged by the need for constant and tireless practice, and they believe that monotony-tolerance (Gr = 120) is necessary for success. The last block of frequency of mentions included decision-making (Gr = 116), creative problem-solving (Gr = 81) and memory (Gr = 30). Decision-making was also considered by respondents to be one of the basic cognitive skills as they considered it unimaginable to play successfully and this was also responsible for the lower frequency. In contrast, creative problem-solving, which was interpreted as a higher-level skill, was indeed not mentioned as often in our interviews, but there was hardly an interview where it was not mentioned. Similarly, in the case of memory, we found that the overall knowledge of the game, the recall of knowledge about the game (to which we also linked references to game intelligence, as participants mostly referred to this feature concerning knowledge of game mechanics) was also treated as a rather basic feature in most of the discussions. Table 3 summarises the identified psychological factors, their corresponding code frequencies and content examples.

**Table 3:** Relevant content examples matching the identified psychological factors.

Code	Gr.	Gender	Status	Content example
A	532	Female, 19	E-athlete	“In esports, people want a little bit more than that, and that requires a lot of patience, so that if things dont go your way, you dont just fall apart and go back and forth, but you have to be patient and wait until you get to the point where you can make progress.”
B	479	Male, 24	E-athlete	“In addition to that, I think mental composure is one of the most important things because if you get upset about the game, you wont be able to concentrate, you'll make more mistakes, and the more mistakes you make you'll probably lose the game.”
C	426	Male, 23	E-athlete	“At the team level, its definitely a team game, because I much prefer it when everybody knows whats going on, and then we have a nice team fight, or everybody pays attention, lets say, in CS, I dont know, everybody holds their own position, and then we go in tactically, with a much better feeling when the team moves in harmony than when I was alone, I dont know, and beating someone alone.”
		Male, 20	E-athlete	“And the second one is very much dependent on what kind of game youre playing as well, how good a team player you are and how much you can cheer up the atmosphere or the spirit of a team, because that can have an amazing impact on the performance of a team, obviously, apart from the raw, pure game performance.”
D	362	Male, 24	E-athlete	“Well, we have to concentrate on the competition psychologically, I think, and concentrate on it, because you have to take on the feeling that its going to be a competition, which I think is one of the most difficult things in the whole sport, to concentrate on it, and you turn off the outside world and know that you have to concentrate on it for the next hour or two or even more, and you have to be on top of it.”
E	260	Male, 30	E-athlete	“Obviously as a team, I want to achieve my goal, but the goals that I miss come from myself, and then I share them with others who are interested and reward you or maybe find out about you, and then I welcome them to my team.”
F	252	Male, 23	Coach	“Situational awareness is very important, and part of making quick decisions is to have the basic information, the knowledge, in a given situation, and to be able to strategically recognize the situation, and experience is also very important, so that if you find yourself in the same situation again, you can recognize what you knew, what you did first, or what you did altogether?”
G	185	Male, 19	E-athlete	“And the fact that I beat him was an extra motivation, not only that I played him, but I beat him, and that helped me even more to play in such an ecstasy in the other matches.”
H	179	Male, 24	E-athlete	“Its very important when you play in a team to communicate, to be a team player, to understand and tolerate each other, and to listen to each other, and if youre playing an individual sport, you have to be careful not to become too isolated.”
I	176	Male, 21	E-athlete	“Hes spent a lot of hours in simulators, and I think he analyses every single mistake, views it, and tries to improve, because if you think about it, an e-athlete starts, a race, then he analyses what was wrong, what needs to be changed, what doesnt need to be changed.”
J	156	Male, 31	E-athlete	“Very high attention, focus, very good reaction time, thinking, how fast you can think, whether it's these motor things, how fast I can shoot there or you can pull the mouse there.”
K	142	Male, 29	Researcher	“When I was playing Overwatch, its always the patches and I felt very harshly that the ones who can really keep up are the ones who can adapt to the newer patches as quickly as possible, and as much as you can feel the lag if youre really into it, if youre slower to adapt, really, its to notice everything, to get into a virtual environment and to recognize the opportunity that your environment presents.”
		Male, 23	E-athlete	“New styles that you can adapt to, if you ever want to play in a team, it teaches you adaptability.”
L	120	Male, 29	Researcher	“So you can make up your mind that this is the necessary evil, this is the terribly boring thing, this is the exercise, this is the way on.”
M	116	Male, 34	E-athlete	“Here, you have to decide in a fraction of a second, for example, which shot to choose or where to pass the ball, and I think you can always improve to make the right decision as quickly as possible.”
N	94	Male, 23	E-athlete	“In any FPS game you won't be able to get very far without eye-hand coordination, whereas in a Hearthstone game, you can be world number one from the toilet.”
O	81	Male, 28	Researcher	“And that's perhaps where it can show up much better, that with a unique solution, a new strategy and so on.”
P	61	Male, 28	Researcher	“Well, one of the things I can say is concentration and divided attention, which I would say is mainly in strategy games, like Starcraft 2, which is an esports game.”
R	30	Male, 22	Coach	“In Dota 2, for example, memorising these things and looking for correlations so that, if one person now, for example I have to know what hero I'm going to click on, then I have to know what items Im going to buy, and I need a very good memory to remember every single little pro thought, I'm going to click on this because this and that are better, Im going to do this and that, I think they can give you more than memory skills and they can see the whole thing in its entirety, so to speak, what were doing and why.”

Source: PTAE database^4^.

### 3.2 Top five list

We considered it important not only to deduce from the content of the focus group discussions and interviews the psychological factors that matter most, as expressed by our participants but also to have the participants create a list of the five most important characteristics for a successful career in esports. At the top of this list (1st place) was the psychological factor considered the most important, while at the bottom (5th place) was the factor considered the least important but still essential. In analysing this question, we treated the focus group discussions separately from the interview discussions, as it was observed during the focus group that the participants influenced each other in the development of the list.

Overall, the most frequently mentioned code in the focus group discussions was the reaction time (Gr = 12), followed closely by eye-hand coordination and resilience (Gr = 11). These were followed by cooperation (Gr = 10), but there were also more than average references to stress tolerance (Gr = 7), adaptability (Gr = 6) and goal-orientedness (Gr = 5). Sustained attention, communication skills, situational awareness and insight, memory, monotony tolerance, and achievement motivation also continued to rank with the same frequency (Gr = 4). The codes with the lowest frequency were decision-making, critical thinking, creative problem-solving and simultaneous attention capacity (Gr = 2).

In the focus group discussions, the most frequently number 1 ranked characteristics were resilience (Gr = 4) and adaptability (Gr = 3). Although resilience was ranked second with the highest frequency, stress tolerance was also mentioned with a high frequency. The third most agreed-upon code was reaction time (Gr = 8), which was often followed by eye-hand coordination (Gr = 4), followed by memory (Gr = 3). Social skills, such as cooperation and communication skills, were ranked fourth with higher frequency (Gr = 3), but also eye-hand coordination was ranked higher than average (Gr = 3). Finally, in fifth place, only goal-orientedness was mentioned more than the average (Gr = 3) (Table 4).

**Table 4:** Coded frequencies of the five characteristics considered most important by participants according to their position in the list (focus group discussions).

Code name	1st place	2nd place	3rd place	4th place	5th place
SUS:	4	3	1	1	2
STRESS:	3	1	1	1	0
COM:	2	2	1	3	2
ACH:	1	1	0	0	0
RES:	1	0	0	1	3
ADA:	1	0	0	1	0
DEC:	1	1	0	1	1
CREA:	1	3	1	1	1
CRI:	1	1	8	1	1
SIT:	1	1	4	3	2
GOAL:	0	0	1	0	1
SIM:	0	1	1	0	0
MON:	0	1	0	1	2
COOP:	0	0	1	1	2
MEM:	0	2	1	1	0
REA:	0	0	3	1	0
EYE:	0	0	1	3	0

Source: PTAE database^5^.

The analysis of the interviews showed that the results were consistent with the focus group discussions. Overall, resilience was mentioned most often (Gr = 30), followed by stress tolerance (Gr = 21), cooperation (Gr = 19), goal-orientedness (Gr = 15) and sustained attention (Gr = 12). Although not as high as those mentioned above, monotony-tolerance (Gr = 11), adaptability (Gr = 10), achievement motivation (Gr = 10), communication skills (Gr = 9) and critical thinking (Gr = 8) were more frequently referred to by our interviewees when creating the ranking list. Finally, our interviewees ranked reaction time (Gr = 6), situational awareness and insight (Gr = 5), eye-hand coordination (Gr = 4), decision-making (Gr = 3), memory (Gr = 3), simultaneous attention (Gr = 2) and creative problem-solving (Gr = 1) with a lower frequency or mentioning them only once or twice.

Stress tolerance (Gr = 8) and resilience (Gr = 5) were the most frequently ranked, but adaptability and cooperation were also mentioned with above-average numbers. In the second place, this order was reversed, as resilience was (Gr = 8) most frequent, followed by stress tolerance (Gr = 5). Nevertheless, characteristics indicating goal-orientedness, critical thinking and achievement motivation also appeared in second place with considerable frequency (Gr = 3). In third place, resilience leads the ranking, followed by social skills (cooperation: 4, communication skills: 3) and adaptability (Gr = 3). In fourth place, the proportions were much more even. The fourth most frequently ranked code (Gr = 5) was goal-orientedness. Stress tolerance, cooperation, monotony-tolerance and achievement motivation were mentioned by our interviewees with the same frequency (Gr = 4) in the fourth place. Finally, in 5th place, cooperation (Gr = 7) and resilience (Gr = 6) were mentioned much more frequently than the average. We then found that, although less frequent than in the other codes, our interviewees referred to sustained attention (Gr = 4), goal-orientedness and critical thinking (Gr = 3) with a higher frequency (Table 5).

**Table 5:** Coded frequencies of the five characteristics considered most important by participants according to their position in the list (interviews).

Code name	1st place	2nd place	3rd place	4th place	5th place
STRESS:	8	5	2	4	2
RES:	5	8	8	3	6
ADA:	4	0	3	2	1
COOP:	3	1	4	4	7
SUS:	2	2	2	2	4
GOAL:	2	3	2	5	3
MON:	2	1	2	4	2
REA:	2	2	1	0	1
CRI:	1	3	1	0	3
ACH:	1	3	1	4	1
EYE:	1	2	0	0	1
DEC:	1	0	1	1	0
COM:	0	2	3	2	2
MEM:	0	0	1	0	2
SIM:	0	0	0	1	1
SIT:	0	1	3	1	0
CREA:	0	1	0	0	0

Source: PTAE database.

Drawing from all these results, three approaches were used by our focus group and interview participants to generate the top five lists. In the case of the first and most commonly used method, they approached the ranking from the perspective of resilience and stress tolerance, followed by social, cognitive and mechanical skills essential for play. Secondly, the focus was on social skills, followed by resilience and stress tolerance, and finally cognitive and mechanical skills. The least used approach was to put cognitive and mechanical abilities first, followed by resilience and stress tolerance, and social skills last.

### 3.3 Group structure of codes, or the frequency of co-occurrence of codes

We analysed our research question to see how our codes were related to each other, whether they could be further clustered, and if so, based on what logical ordering. To answer these research questions, Code Co-occurrence analysis was run in ATLAS.ti.

Examining adaptability, we found that critical thinking, situational awareness and insight, and cooperation had the highest joint frequency. Regarding goal-orientedness, three codes also showed a higher joint frequency: achievement motivation, resilience, and cooperation. Decision-making ability had a lower frequency as mentioned above, but still showed a combined frequency of over one hundred with reaction time, situational awareness and insight. Cooperation showed a higher co-occurrence with resilience and communication skills. The latter showed a much higher co-occurrence rate than the former, suggesting that cooperation is closely related to communication skills. Considering sustained attention, it was clear that it was mentioned most frequently with stress tolerance, but also showed a high co-occurrence with resilience. When we looked at situational awareness and insight, we found that this had the highest frequency with critical thinking ability, but a frequency above 100 was observed with stress tolerance, reaction time and creative problem-solving. Memory was low in frequency numbers, but half content related to this code co-occurred with situational awareness and insight. The communication skills code did not show a particularly high co-occurrence rate with anything other than cooperation. Considering creative problem-solving ability, it was apparent that it had a particularly high co-frequency value with criticalthinking. Regarding critical thinking, in one-third of the mentions, it went hand in hand with resilience. Continuing with monotony-tolerance, we again found a close co-occurrence with resilience. Already during the conversations, the close relationship between reaction time and eye-hand coordination was clear from the constant emphasis of our participants, and this was confirmed by the analysis, as in half of the cases these two codes co-occurred. Our most frequently identified code, resilience, was mentioned with stress tolerance in the highest proportion of cases but also showed high frequency with achievement motivation. Finally, observing stress tolerance, our analysis was complemented by the higher co-occurrence of the aforementioned code and achievement motivation (Table 6).

**Table 6:** Code co-occurrence frequencies.

Code name	Ach Gr = 425	Sim Gr = 86	Eye Gr = 141	Str Gr = 723	Res Gr = 893	Rea Gr = 204	Mon Gr = 231	Mem Gr = 90	Cri Gr = 364	Cre Gr = 179	Com Gr = 293	Sit Gr = 441	Sus Gr = 472	Coop Gr = 692	Dec Gr = 192	Goal Gr = 471	Ada Gr = 237
Ada Gr = 237	26	0	12	37	50	29	13	25	102	63	44	109	10	101	32	24	0
Goal Gr = 471	176	3	12	51	249	11	51	1	54	12	19	41	72	123	9	0	
Dec Gr = 192	22	14	16	69	32	125	8	17	66	20	26	162	60	63	0		
Coop Gr = 692	100	42	12	134	184	48	21	20	88	69	362	152	72	0			
Sus Gr = 472	62	60	30	228	187	51	49	21	67	32	20	127	0				
Sit Gr = 441	69	50	52	110	81	111	23	41	250	105	69	0					
Com Gr = 293	18	10	6	64	59	21	17	14	55	53	0						
Cre Gr = 179	25	7	9	45	52	21	13	20	127	0							
Cri Gr = 364	55	24	16	79	111	30	28	43	0								
Mem Gr = 90	8	18	32	33	32	45	11	0									
Mon Gr = 231	17	6	20	28	190	18	0										
Rea Gr = 204	4	15	69	24	61	0											
Res Gr = 893	176	29	47	378	0												
Str Gr = 723	103	24	36	0													
Eye Gr = 141	4	5	0														
Sim Gr = 86	13	0															
Ach Gr = 425	0																

Source: PTAE database.

Based on our knowledge of the literature and the results of our analysis, we were thus able to identify six clusters of mental characteristics and skills relevant to e-athletes.

The first cluster we called Developmental motivation, which includes the characteristics of goal-orientedness and achievement motivation, as these help to set the e-athletes on a path to progress, motivate them to improve and serve as a motivational base to draw on in difficult times (e.g., through recalling previous successful situations and achievements).

The second is the cluster of Attentional Skills, which includes sustained attention and simultaneous attentional capacity. It includes the cognitive abilities that allow long-term concentration on the game, filtering the slightest changes in the observed image, and dividing attention depending on the game title.

The third and most comprehensive cluster we called Game Intelligence. These included adaptability, decision-making, situational awareness and insight, creative problem-solving, critical thinking and memory. These mental characteristics and cognitive abilities are most appropriately highlighted and exploited through the game that is played. Based on this, the comprehensive knowledge of a given game, the ability to recall as quickly and as accurately as possible the knowledge related to it, the ability to adapt to changes in the situations affecting and occurring within the game, the ability to make decisions based on these changes, to make decisions appropriate to the strategy and tactics used by the situation and the opponent, to recognise and learn from one's own mistakes, to think up new strategies and tactics, to improve existing ones, and the ability to conclude about the other's game and strategy can all be included here.

The fourth cluster is Perseverance and stress management, which includes monotony-tolerance, resilience and stress tolerance. On the one hand, it refers to the appropriate endurance during continuous, monotonous exercises, as well as coping with the stress experienced in the short and long term, and the appropriate management of the experience of success and failure. Problems in these areas, such as competitive overwork and anxiety, can have a direct impact on cognitive abilities (e.g., reduced efficiency of attention and concentration).

Motor Skills form the fifth cluster, which includes eye-hand coordination and the reaction time that strongly influences it. These mainly affect the speed and precision with which e-athletes can deliver instructions as inputs to the game. These enable the e-athletes to make good use of the characteristics and skills included in the Game Intelligence cluster.

The final cluster is the Social Skills, which includes adaptability, cooperation and communication skills. These skills are essential for working effectively with coaches and other members of the sports organisation, as well as with teammates in team e-sports.

Adaptability was the only skill that was classified into two clusters. It was because respondents also approached this ability from two main perspectives. E-athletes need to be able to adapt to changes in the game and emerging game situations, alternatively, from a social point of view, it was also understood as adapting to the coach, the team or new teammates joining the team.

## 4 Discussion

The results suggest that the performance of e-athletes could be influenced by several psychologically relevant factors. 17 characteristics were identified and references were sought during the coding process. The code with the highest frequency was resilience, which, according to the participants, is a key component for becoming a professional e-athlete and being able to have a successful career. This further confirms the research findings of Himmelstein and colleagues (2017). All other characteristics are based on it or make sense because of it. This was explained by one participant as follows:

“One example is that my friend... who everyone was amazed at what a genius he was, but he disappeared from the game, I don't know where he went. So it’s so easy for players to disappear these days if they don't pay more attention to the game. At least I feel like it’s easy for people to get sidetracked like it would be cool to play that Indie game or something. We have seen specific examples of young talents really disappearing from the surface and it may be simply because they have not consciously managed certain attitudes or even if they should have been made aware of things that are not game-specific and which can help a lot in their development otherwise.”

(Male e-athlete, 25)

Other factors that consistently drove performance were goal-orientedness and a achievement motivation built strongly on resilience to help e-athletes. The results suggest that some factors are less generalisable, or at least of different importance and use in several game titles. Examples include outstanding reaction time and associated eye-hand coordination, which are more pronounced in esports game genres such as shooters or MOBAs, and less pronounced in card games and turn-based games (63). Social skills such as the ability to adapt to others, cooperation and communication are much more crucial in team esports games than in individual ones (49, 56, 59–61). On the other hand, there are identified factors, such as stress tolerance and sustained attention, which are consistent, regardless of game genre, but not to the same extent as resilience, determinants of esports performance (65, 76). To a lesser extent, game-related adaptability, monotony tolerance and simultaneous attention capacity were also found to be important. Finally, critical thinking, situational awareness and insight, decision-making, creative problem-solving, and memory skills were the top esports performance skills for solving strategic or unexpected situations in the game (37, 53–57). In many cases, there were examples at the end of the interviews of characteristics such as reaction time, simultaneous attention capacity and eye-hand coordination being referred to as such relevant characteristics that it did not even occur to them to mention them separately.

Using all these characteristics six clusters were identified: Developmental Motivation, Attentional Skills, Game Intelligence, Perseverance and Stress Management, Motor Skills, and Social Skills. These clusters can also be interpreted as groups of competencies, into which the characteristics described and used in the analysis above have been grouped. It is also important to highlight the characteristics that are part of Game Intelligence, such as the acquisition and recall of game-specific knowledge, the recognition of situations based on this knowledge, the appropriate decision-making and the drawing of further conclusions, which were treated similarly by the interview subjects as eye-hand coordination, simultaneous attention capacity and reaction time. These clusters could also help intervention and training programs (e. g. mindfulness training, goal setting and motivation training, etc.) become more tailored to the esports context by highlighting specific needs. This could be helpful for these practices to be more focused on real-world performance challenges, making them more relatable and impactful for e-athletes. These intervention and training programs would also be beneficial to support e-athletes to become elite esports players while overcoming challenges related to dual-career (43, 70), and to overcome gender disparities in esports (42).

Overall, the performance of e-athletes can be influenced by a wide range of psychological characteristics. Among these factors, some are more generalisable and of almost equal importance, in the case of the present research, these were resilience, stress tolerance and sustained attention. However, there are characteristics of each esports genre as well as whether the esports title is an individual or team sport with varying emphasis. For example, cooperation and communication skills, are of course also important when working with a coach in individual esports, but can have a greater impact in team esports. The characteristics used in the analysis of the research were classified into six distinct groups of competencies, which along these lines have different potential to influence performance in esports.

### 4.1 Strengths and limitations

Considering the practical utility of the research, we have identified psychologically relevant factors that can influence e-athlete performance and have developed clusters along these factors, which can be considered competence groups. These characteristics and competency groups can also be used to assist in e-athlete and youth selection assessments and to guide the development of psychological tests that meet esports-specific scientific standards, thus helping to improve the quality of psychodiagnostic work with e-athletes.

Limitations of this research include the fact that the proportion of representatives from esports titles cannot be considered representative and that we could not interview esports players from every esports title. Moreover, the present research sample was composed of Hungarian participants, so an increase in the sample in terms of quantity and internationalisation would have significantly increased the generalisability of the results. In addition, Hungarian cultural attitudes toward gaming and esports as a whole influenced how participants perceived their participation in esports. Many felt like they needed to validate their engagement due to societal scepticism, particularly scepticism coming from older generations. This aligns with local norms that traditional career paths are still more preferred over emerging digital professions like esports. A further limitation is that motor skills such as eye-hand coordination, the reaction time behind quick decision-making, and the characteristics that make up the Game Intelligence competence group were not necessarily interpreted as psychological or mental characteristics, so fewer references were verbalised for them.

### 4.2 Later research

The clusters and characteristics as determinants of esports performance could serve as a basis for further research that could contribute to a more detailed and comprehensive understanding of esports and performance in both overall and genre- and even game title-specific studies. In addition, individual and team esports comparisons can be used as a guide to identify similarities and differences, as well as the challenges they usually face.

## 5 Conclusion

The present research aimed to explore and confirm the findings of previous research on the psychological characteristics that can influence performance in esports. To this end, we used a qualitative methodology in the form of focus group discussions and interviews with e-athletes and people with relevant experience and insight into esports. Using 17 different characteristics, the content of the conversations was analysed, with three coders taking part in the analysis. As a result of the analysis, the most prominent characteristics that were most frequently referred to were resilience, stress tolerance, cooperation and sustained attention. Furthermore, we used the results of the analysis to create six clusters, which we interpreted as competence groups. As a result of the research, it was possible to identify and categorise characteristics and competencies that can serve as a basis for future psychodiagnostic assessments of e-athletes, for the development of matching tests, and as a reference for selection and talent management.

## Data Availability

The raw data supporting the conclusions of this article will be made available by the authors, without undue reservation.

## Footnotes

^1^**Explanation of the signs:** TBS, turn-based strategy games; AB, auto-battler games; CG, card games; RTS, real-time strategy games; MOBA, multiplayer online battle arena games; DTS, digital traditional sports games; HS, hero-shooter games; OS, other simulation games; SFE, sports games with fantasy elements; EX, exergames; FPS, first person shooter games; BR, battle royal games; TPS, third person shooter games; FG, fightning games; MMORPG, massively multiplayer online roleplaying games.

^2^Age of Empires 4, Brawl Stars, Counter-Strike: Global Offensive, Dota 2, Fifa, Formula 1, Gwent: The Witcher Card Game, Hearthstone, Heroes of the Storm, League of Legends, Overwatch, Paladins, Player Unknown’s Battleground, Rocket League, Teamfight Tactics.

^3^In Hungarian, words can be broken down into constituent elements (morphemes). A morpheme is the smallest unit of a word that has an independent form and meaning. In the Hungarian language, suffix morphology and verb conjugation can significantly alter word forms, which could affect computer processing (74). Thus in our research, since the interviews were recorded in Hungarian we also looked for the most commonly used substituted morphemes.

^4^**Explanation of terms:** (A) resilience; (B) stress-tolerance; (C) cooperation; (D) sustained attention; (E) goal-orientedness; (F) situational awareness and insight; (G) achievement motivation; (H) communication skills; (I) critical thinking; (J) reaction time; (K) adaptibility; (L) monotony-tolerance; (M) decision making; (N) eye-hand coordination; (O) creative problem-solving; (P) simultaneous attention capacity; (R) memory.

^5^Note: Colours are used to indicate the codes with the highest observed frequency at a given location. In cases where a code was mentioned by three or more people in the same location, it was interpreted as an above-average frequency.
